# Identifying the fitness costs of a pyrethroid-resistant genotype in the major arboviral vector *Aedes aegypti*

**DOI:** 10.1186/s13071-020-04238-4

**Published:** 2020-07-20

**Authors:** Lisa M. Rigby, Gordana Rašić, Christopher L. Peatey, Leon E. Hugo, Nigel W. Beebe, Gregor J. Devine

**Affiliations:** 1 Australian Defence Force Malaria and Infectious Disease Institute, Gallipoli Barracks, Enoggera, QLD 4051 Australia; 2 Mosquito Control Laboratory, QIMR Berghofer Medical Research Institute, Herston, QLD 4006 Australia; 3 School of Biological Sciences, University of Queensland, Brisbane, Australia; 4 CSIRO, Brisbane, QLD Australia

**Keywords:** *Aedes aegypti*, Pyrethroid, Insecticide resistance, Backcross, Timor-Leste

## Abstract

**Background:**

Effective vector control measures are essential in a world where many mosquito-borne diseases have no vaccines or drug therapies available. Insecticidal tools remain the mainstay of most vector-borne disease management programmes, although their use for both agricultural and public health purposes has resulted in selection for resistance. Despite this, little is known about the fitness costs associated with specific insecticide-resistant genotypes and their implications for the management of resistance. In *Aedes aegypti*, the primary vector of dengue, chikungunya, and Zika, the best-characterised resistance mechanisms are single-point mutations that protect the voltage-gated sodium channel from the action of pyrethroids.

**Methods:**

We evaluated the fitness cost of two co-occurring, homozygous mutations (V1016G and S989P) by back-crossing a resistant strain of *A. aegypti* from Timor-Leste into a fully susceptible strain from Queensland. The creation of the backcross strain allowed us to isolate these kdr mutations in an otherwise susceptible genetic background.

**Results:**

In comparison to the susceptible strain, the backcrossed colony exhibited longer larval development times (5 days, *P* < 0.001), 24% fewer mosquitoes reached the adult stage (*P* = 0.005), had smaller wing lengths (females, *P* = 0.019 and males, *P* = 0.007) and adult female mosquitoes had a shorter average lifespan (6 days, *P* < 0.0006).

**Conclusions:**

These results suggest specific and significant fitness costs associated with the double homozygous V1016G/S989P genotype in the absence of insecticides. The susceptibility of a population may recover if the fitness costs of resistant genotypes can be emphasised through the use of insecticide rotations and mosaics or the presence of untreated spatial or temporal refuges. 
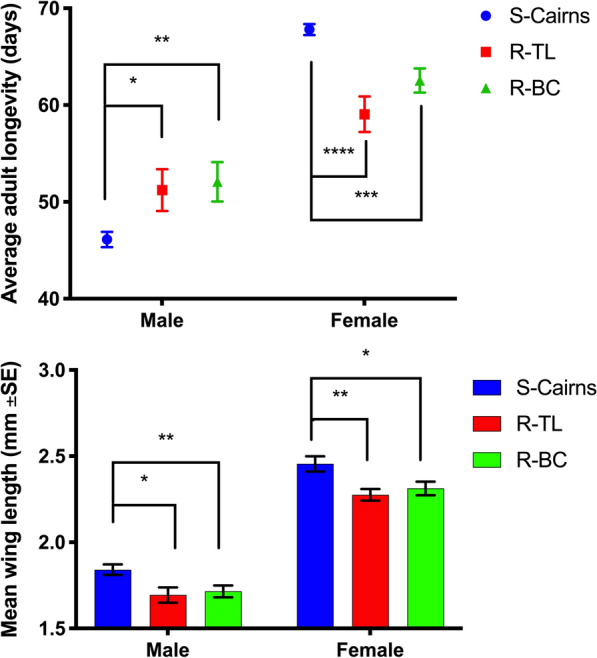

## Background

The implementation and efficacy of vector control measures are essential in a world where many mosquito-borne diseases have no vaccines or drug therapies, but the efficacy of insecticidal tools is challenged by the development of resistance in their targets. Single nucleotide polymorphisms with a functional role in insecticide resistance are now widespread across the globe [1, 2]. These confer significant advantages to mosquitoes in the presence of insecticides, but they are also associated with pleiotropic impacts, which may have a fitness cost in the absence of insecticide exposure [3, 4]. Understanding the association between resistant genotypes and their fitness costs is crucial to the conservation of susceptibility, the development of more effective resistance management programs, and the subsequent maintenance of tools that can be used to manage vector-borne disease.

The heavy use of insecticides in both public health and agriculture has led to the evolution of a variety of resistance mechanisms, including increased metabolic detoxification of insecticides and decreased sensitivity of the target proteins [5]. One of the best-characterised mechanisms of insecticide resistance is knockdown resistance (kdr). The knockdown resistance phenotype is typically described as temporary paralysis of the insect following exposure to a neurotoxic insecticide, followed by complete recovery in mobility [6]. The mechanisms behind kdr are point mutations of the voltage-gated sodium channel (VGSC) gene that confer resistance to dichlorodiphenyltrichloroethane (DDT) and synthetic pyrethroids [7]. The latter represents one of the most effective and widely used classes of insecticides for the management of disease vectors. Alone or in combination with other resistance mechanisms, kdr is associated with fitness costs when insecticides are absent [3, 4, 8, 9].

In *A. aegypti*, the main vector of dengue, chikungunya and Zika viruses, there are four kdr mutations that have been proven to reduce the sensitivity of the VGSC to pyrethroids. These are V1016G, I1011M, V410L and F1534C (using the nomenclature of the housefly VGSC sequence [10–12]). The V1016G mutation is a valine to glycine substitution at position 1016 within domain II of the VGSC. It is often linked to the S989P mutation, a serine to proline substitution at position 989. The S989P mutation has not been found to confer insecticide resistance directly [11]; however, this mutation may play a role in pyrethroid resistance as reports of greater sensitivity to deltamethrin and permethrin have been recorded in instances where this mutation is present in combination with other kdr mutations [13, 14]. The kdr mutation I1011M, an isoleucine to methionine substitution, is also located in domain II of the VGSC gene [11], while the V410L mutation is a valine and leucine substitution in segment 6 of domain I [12]. F1534C is a phenylalanine to cysteine substitution at position 1534 in domain III, segment 6 [11]. The V1016G kdr mutation is widely distributed across Asian *A. aegypti* populations [15, 16] and has also recently been documented in *Aedes albopictus* [17, 18]. Alone, or in combination with other mutations such as F1534C [13, 17, 18], these alleles confer significant protection against pyrethroid insecticides [11].

In *A. aegypti,* the impact of kdr mutations and metabolic mechanisms has been investigated for several life-history traits [3, 4, 19]. These studies reported kdr-associated reductions of egg hatchability, increased larval development times, reduced adult female body size, and reduced female fecundity; however, these costs are not consistent among studies. In comparison with susceptible individuals, *A. aegypti* harbouring the F1534C mutation were found to have smaller female wing lengths, but larval development, pupation success, adult emergence and mating ability were unaffected [19]. In contrast, the V1016G/S989P genotype in *A. aegypti* was associated with prolonged larval development, reduced size and reduced hatching rate [4]. Very few studies on *A. aegypti* have assessed kdr mutations in isolation from other resistant mechanisms and all are potentially confounded by the additional impacts of other unidentified alleles with unknown effects. Therefore, the specific fitness costs of kdr genotypes alone remain largely unresolved. The one exception to this is a study that used backcrossing to isolate homozygous forms of the mutations V1016I and F1534C in an otherwise susceptible background [3]. Given that V1016I has little impact on resistance against pyrethroids [10], the increased larval development times, reduced fecundity and weak competition in caged populations found in that study, were ascribed mainly to the functional resistance caused by F1534C [3].

In this study, we backcrossed a resistant strain of *A. aegypti* from Timor-Leste, containing the V1016G and S989P kdr mutations, with a fully susceptible strain from Queensland, Australia. The result was a colony homozygous for the two point mutations but whose genetic identity was close to that of the susceptible parent. To ensure the retention of kdr, we repeatedly selected the backcrossed progeny with a diagnostic dose of a synthetic pyrethroid. We confirmed the susceptible genetic background in our backcross strain by screening genome-wide variation using double-digest restriction associated sequencing (ddRAD-seq). This meant that observed differences in life-history traits between the backcrossed-resistant and susceptible parental strain could be attributed to the pleiotropic effects of the homozygous V1016G/S989P genotype.

## Methods

### *Aedes aegypti* strains

Two strains of *A. aegypti* were used as the parental strains of our backcross. R-TL is a pyrethroid-resistant strain that originated from Dili, Timor-Leste, in 2009 [20]. S-Cairns is an insecticide susceptible reference strain of *A. aegypti* collected in Cairns, Australia, in 2015. From these parent colonies, we created a backcrossed strain of *A. aegypti* (R-BC) that carries the R-TL kdr mutations in an S-Cairns genetic background (as described below). All mosquitoes used in this study were maintained using standard laboratory protocols, previously described [21]. Mosquitoes were blood-fed *via* an artificial membrane feeding system and defibrinated sheep blood (Serum Australis).

### Introduction of pyrethroid resistance mechanisms into the susceptible genomic background

The method used to create the R-BC strain was repeated backcrossing with selection [8, 22, 23]. Initially, the offspring from isolated pairs (♀ R-TL x ♂ S-Cairns) were selected for insecticide-resistant phenotypes using the diagnostic dose (DD) and exposure time for the synthetic pyrethroid, permethrin, according to CDC protocols [24]. Surviving F1 female individuals were then paired with S-Cairns males. Using a minimum of five surviving females at each generation, this procedure continued to F12 after which time the surviving progeny were allowed to mate with siblings and selected for resistance to increase homozygosity for the kdr alleles (Additional file 1: Figure S1). From F18 onward, the backcrossed individuals (R-BC) displayed a resistant phenotype comparable to the resistant R-TL parental strain (Additional file 2: Figure S2). The V1016G and S989P target-site mutations were present in a homozygous state in 100% of tested R-BC individuals.

### Insecticide resistance characterisation in *A. aegypti* strains

The insecticide resistance status of S-Cairns, R-TL and R-BC strains was determined using the CDC bottle bioassay. Ten to 20 unfed, two-5 day old *A. aegypti* females were introduced to Wheaton bottles coated with insecticide or acetone only (control), following the CDC bottle bioassay protocol and DD [24]. Three pyrethroids (permethrin, deltamethrin and lambda-cyhalothrin), one organophosphate (malathion), one organochlorine (DDT) and a carbamate (bendiocarb), were tested. The number of mosquitoes that were “knocked down” (KD; unable to fly or right themselves when the bottle is gently rotated), dead or alive, were monitored at 15-min time intervals (0–120 min) post-exposure. To fully characterise the phenotype, dose-response assays for permethrin were also performed at a range of multiples of the DD (15 µg, 30 µg, 75 µg, and 150 µg/bottle). Statistical differences in the dose-response curves were calculated using area under the curve (AUC; GraphPad Prism version 7.00). To test for the presence of metabolic resistance mechanisms, we used the CDC protocol with the addition of piperonyl butoxide (PBO) as an oxidase inhibitor and diethyl maleate (DM) as a glutathione transferase inhibitor [24] at the concentrations specified by the CDC protocol [24]. Mosquitoes were exposed to bottles coated with synergists for 60 min before transfer to bottles containing the permethrin DD. Time to KD was monitored as above, and difference in proportion of KD mosquitoes was tested using the two–proportion z–test.

### Genotype characterisation

#### Screening of kdr mutations

Genomic DNA was isolated from 30 randomly collected individuals from the parental strains and then from the backcross generation once the phenotype consistently reflected the resistant parent (F23), using a Qiagen Blood and Tissue extraction kit (Qiagen, Hilden, Germany, Cat No: 69506). We amplified two parts of the VGSC gene (VectorBase gene ID: AAEL013277). One amplicon encompassed mutations at positions 989, 1011 and 1016 in domain II using the primers AaSCF1 and AaSCF4 [25]. The second amplicon covered position 1534 and used the primers AaSCF7 and AaSCR7 [25]. Both amplicons per individual were analysed using Sanger sequencing conducted by the Australian Genome Research Facility (Brisbane, Australia). Sequences were analysed using the software, Geneious, version 9.1.4 (1) (http://geneious.com). Genotypes were identified according to the presence of clear singular peaks for glycine, proline, or cysteine at the positions 1016, 989 and 1534, respectively.

#### Screening of genome-wide variation using double-digest restriction associated sequencing (ddRAD-seq)

Genome-wide variation in individuals from our three strains was screened using ddRAD sequencing as described in Rašić et al. [26]. The final dataset contained 35 individuals from the susceptible parent (S-Cairns), 15 individuals from the resistant parent (R-TL) and 25 individuals from the backcross colony (R-BC) whose phenotype reflected the resistant parent (F23 generation). Briefly, libraries were generated using 100 ng of total genomic DNA per individual, extracted using the Qiagen Blood and Tissue kit (Qiagen, Cat No: 69506) and digested using NlaIII and MluCI restriction enzymes (New England Biolabs, Beverly, MA, USA). The resulting digestions were cleaned using SPRI magnetic beads, and fragments were barcoded using modified Illumina P1 and P2 adapters arranged in a scheme that enables the unique identification of individuals. The ligations were pooled and cleaned with SPRI magnetic beads. Fragments between 300–450 bp were size selected with the Blue Pippin 2% agarose kit (Sage Sciences, Beverly, MA, USA). PCR was performed on the fragments with Illumina primers. Final libraries were sequenced with an Illumina HiSeq 4000 using a 100-bp pair-end.

#### Genomic data processing and analysis

Paired-end FASTA files for each individual were obtained by demultiplexing the raw files with DDemux [26]. The FASTA files were run through the pipeline developed by Rašić et al. [26], utilizing the trim_and_align.sh shell script, used to retain high-quality reads (Phred > 20) and trimmed to 90 bp. The resulting high-quality sequences were aligned on the latest improved genome assembly AaegL5 [27] using Bowtie 1.2 [28]. Genotype calling was completed using the Stacks pipeline with minimum stack depth of five and VCF files were generated with the *populations* module [29]. VCFtools (v0.1.12b) [30] was used to remove single-nucleotide polymorphism (SNP) loci if they were absent in more than 25% of individuals from each of the three population samples and if the minor allele did not occur in at least two individuals across all samples. SNPs were assigned to chromosomes, according to Matthew et al. [27] and the numbers recovered from each of the datasets are shown in Additional file 3: Table S1. The proportion of the genome originating from each of the parental strains (R-TL and S-Cairns, K = 2) that was present in each individual from the backcrossed strain (R-BC) was assessed using ADMIXTURE analysis [31]. To remove SNPs in high linkage disequilibrium, we pruned the initial dataset using–indep-pairwise option of PLINK. VCFtools (v0.1.12b) was also used to calculate heterozygosity per individual and pairwise *F*_ST_ between the strains (Weir and Cockerham’s AMOVA-based method) using the 5 Mbp moving-window average for each chromosome [26].

### Evaluating the impact of resistance on fitness traits

All measured fitness components (fecundity, larval development time, pupae formation, adult longevity, and adult size) were compared between the parental strains (S-Cairns and R-TL) and the backcrossed strain (R-BC) under identical environmental conditions (see: *A. aegypti* strains). Comparisons were conducted in a single controlled temperature room at QIMR Berghofer Medical Research Institute, maintained at 27 ± 1 °C, 70% humidity, and a 12:12 h day:night light cycle. All data on fitness traits were analysed in R3.01, except for the survival analysis, which was completed in GraphPad Prism version 7.00.

#### Fecundity

Fifty newly emerged males and 50 newly emerged unmated females (< 12 hour-old) were confined in a BugDorm-1 (30 × 30 × 30 cm, BugDorm Store, Taichung, Taiwan) for 3 days to allow mating before being offered a blood meal. One day after blood-feeding, 15 fully engorged female individuals from each strain were randomly selected and each was confined to a 25 ml container lined with filter paper and cotton wool. Twenty-four hours after blood-feeding, the filter paper and cotton wool were moistened with 5 ml of de-chlorinated water and females were confined for an additional 72 h. We recorded the number of females that did not produce eggs, and the number of eggs per female for those that laid at least one egg (non-zero data). Fecundity experiments were completed in triplicate. The Chi-square test for independence was used to test the between-strain difference in the proportion of females that laid eggs. Egg count per female (non–zero data) was tested for normality using the Anderson–Darling test, and difference between strains was tested using Kruskal–Wallis ANOVA and pairwise Mann–Whitney test.

#### Larval development time and pupae formation

Fifty newly emerged larvae (undetermined male and female ratios, < 12 hours-old) were transferred to plastic trays (33 × 24 × 8 cm) containing 5 l of de-chlorinated tap water. Larvae were given 0.5 g of Tetramin fish food every 48 h. This is a low density, high nutrition regime that aims to maximise larval survival and reduce competition. Total larval development time was measured as the number of days between hatching and pupation. Larvae were counted daily. All individuals molting to the pupal stage were recorded and removed. Emerged adult mosquitoes were also counted and sexed. Experiments were performed in triplicate. The Chi-square test for independence was used to test the between-strain difference in the proportion of larvae that survived to the adult stage. Differences in the larval development time were calculated using the Log–rank (Mantel–Cox) test, and differences in the median number of days from first instar to pupae were tested using the Mann–Whitney test. Difference among strains in sex ratio for emerged adult mosquitoes was tested using the Chi-square test.

#### Adult longevity

Adult mosquitoes (< 24 h post-emergence), were confined in a BugDorm-1 (30 × 30 × 30 cm, BugDorm Store, Taichung, Taiwan), supplied with 10% sugar solution ad libitum and monitored daily until death. Male and female adult mosquitoes were observed separately, at a density of 40 per cage (as per rearing methods above). Experiments were repeated three times. Survival curves were compared using the Log-rank (Mantel-Cox) test.

#### Mosquito size

Wing length was adopted as a proxy for mosquito body size [32]. The right wing of each individual was removed, fixed onto slides and photographed using an Olympus SZ40 stereo microscope attached to a Touptek UCMOS Series C-mount USB 2.0 CMOS Camera. Wing length was measured from the tip to the distal end of the alula, excluding the wing fringe, with the software Image J (version 1.52a). All wings were measured twice to account for potential measurement error. At least 45 individuals of each sex, from each strain were analysed. Wing length data from each group (strain, sex) was tested for normality using the Anderson-Darling test and homogeneity of variances using Leven’s test. Differences between groups were tested using the Student’s t-test.

## Results

### Characterisation of phenotypes

To date, insecticide resistance has not been detected in any *A. aegypti* population from Australia [33]. Our Australian strain, S-Cairns was fully susceptible to all tested insecticides (100% mortality) after a 30–45-min exposure to the diagnostic dose (DD) (Table 1).

**Table 1:** The diagnostic concentrations, time points and percentage mosquito survival (Mean ± SE) for each insecticide used in the bioassays with the S-Cairns, R-TL, and R-BC strains

Insecticide	Diagnostic concentration (μg/bottle)	Diagnostic time (min)	%Survival S-Cairns	%Survival R-TL	%Survival R-BC
Permethrin	10	30	0	100	100
Deltamethrin	10	30	0	28 ± 14	14 ±10
Malathion	50	30	0	0	0
Lambda-cyhalothrin	15	30	0	55 ± 19	92 ± 7
Bendiocarb	12.5	30	0	0	0
DDT	75	45	0	100	100

The R-TL and R-BC strains exhibited high resistance to permethrin, with 100% survival after 30 min of exposure. These strains also showed elevated resistance to lambda-cyhalothrin, low-level resistance to deltamethrin and, full susceptibility to malathion and bendiocarb (Table 1). The addition of the enzyme inhibitor PBO significantly increased the impact of insecticides, more so in the parental R-TL strain (*Z* = 4.565, *P* < 0.001) than in the backcrossed strain R-BC (*Z* = 2.345, *P* = 0.01) (Table 2). The addition of DEM also had a significant impact, increasing the lethality of permethrin both in the parental and the backcross strain (R-TL *Z* = 1.695, *P* = 0.045; R-BC *Z* = 3.160, *P* = 0.001). These results indicate that metabolic resistance seems more pronounced in the parental strain, suggesting a loss of certain metabolic resistance components during the backcrossing procedure.

**Table 2:** Percentage survival (Mean ± SE) of R-TL and R-BC strains exposed to the diagnostic concentration of permethrin for 30 min in the presence or absence of PBO or DEM

Insecticide/synergist	Mosquito strain	
	R-TL	R-BC
Permethrin	100%	100%
PBO + Permethrin	76 ± 11%	93 ± 2%
DEM + Permethrin	96 ± 4%	86 ± 4%

**Fig. 1:**
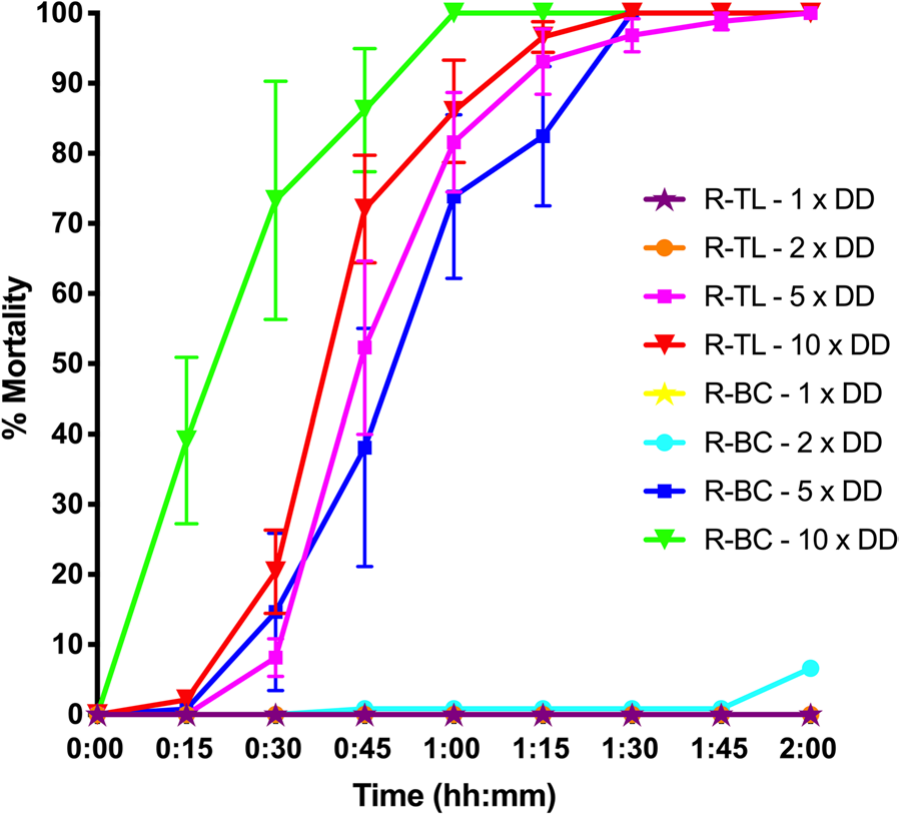
Dose-response curves for R-TL and R-BC in response to 1, 2, 5 and 10 times DD for permethrin for *Aedes aegypti* (Mean ± SE)

The process of backcrossing and selection resulted in the R-BC strain which exhibited a similar, but not identical, phenotype to the parental R-TL strain. Exposure to multiples of the diagnostic dose had little impact on R-TL or R-BC mortality until 10 times the dose when AUC calculations revealed significantly higher mortality (*P* = 0.02) in the R-BC strain in comparison to the parental R-TL strain (Fig. 1). This suggests the loss of some tolerance during the backcrossing process, which could possibly be attributed to differences in metabolic resistance mechanisms between the R-BC and R-TL strains.

### Characterisation of VGSC genotypes

Amplicon sequencing of the partial VGSC gene in the susceptible parental strain S-Cairns revealed only wild-type alleles at positions 989, 1016 and 1534. In the resistant parental strain R-TL, we recorded 100% homozygosity for the resistant kdr mutations S989P and V1016G, and 100% homozygosity for the susceptible wild-type allele at the 1534 position. Likewise, the backcrossed R-BC strain was 100% homozygous for alleles from the resistant parental strain (100% mutations at 989 and 1016, 100% wild-type allele at the 1534 position), once it displayed a similar resistant phenotype to the R-TL parental strain and subsequently deemed suitable for use in analysing fitness traits (generation F23, Additional file 2: Figure S2).

### Comparison of genetic backgrounds

**Fig. 2:**
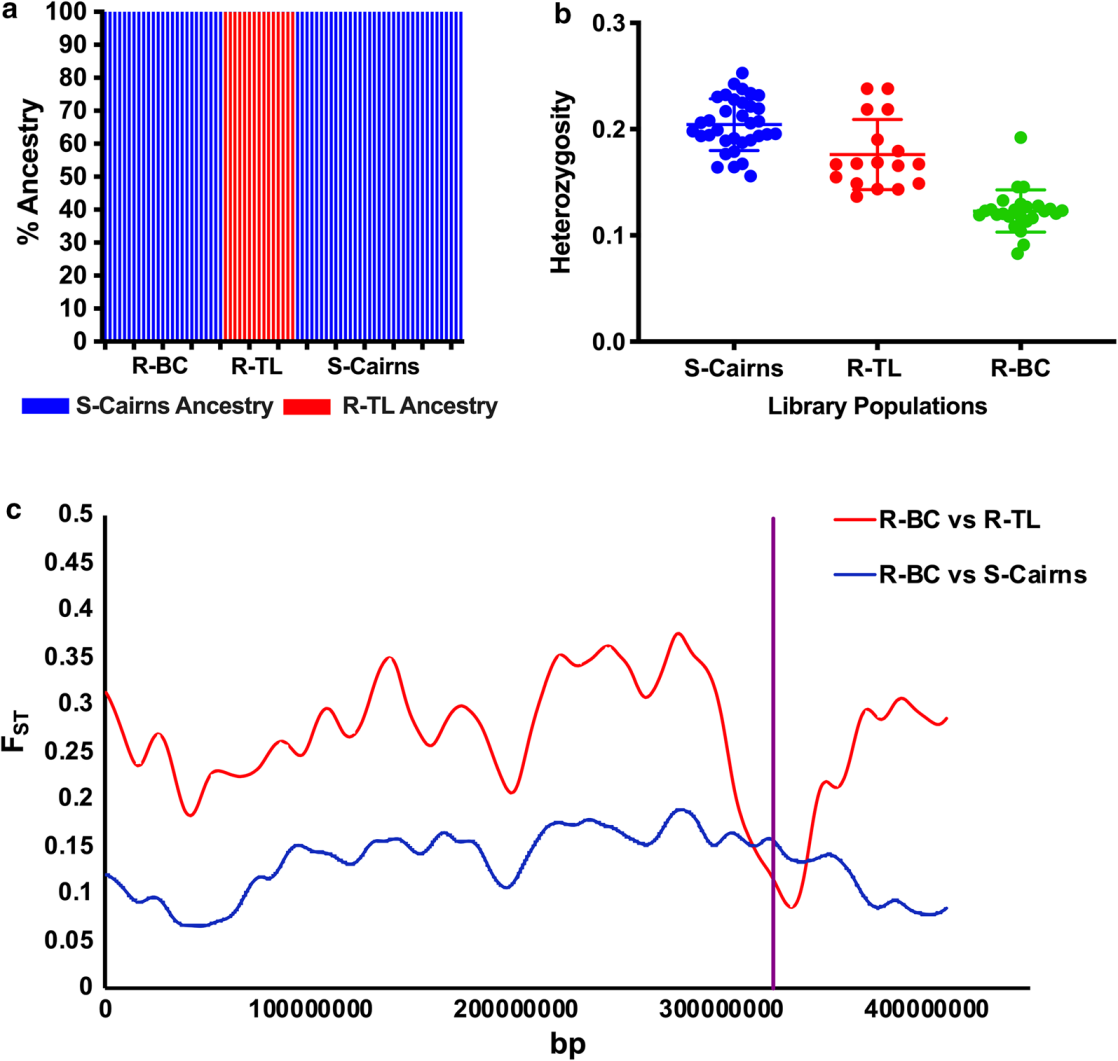
**a** Analysis of genome-wide variation Ancestry fraction (Q) estimate plot for each of the three *Aedes aegypti* strains from ADMIXTURE analysis for K = 2. **b** Per-individual heterozygosity level in the three strains (Mean ± SE). **c** Pattern of allele frequency differences between R-BC and R-TL (red) and R-BC and S-Cairns (blue) (measured as per-locus pairwise *F*_ST_) along chromosome 3, calculated over a 5Mb moving-window average. The vertical purple line approximates the chromosomal position of the VGSC gene

Analysis of genome-wide variation and structuring revealed that each individual in the backcrossed strain (R-BC) had > 99.9% of its genome originating from the susceptible parental strain, S-Cairns (as demonstrated by the Q plot from an ADMIXTURE analysis, assuming two source populations, K = 2, Fig. 2a). Mosquitoes from the backcrossed strain also had lower genome-wide heterozygosity than mosquitoes from the parental strains (Fig. 2b). Genome-wide *F*_ST_ (differentiation) was lower between the backcross and susceptible strain (R-BC vs S-Cairns *F*_ST_ = 0.098) than between the backcross and resistant strain (R-BC vs R-TL *F*_ST_ = 0.237, Table 3). The only genomic region where R-BC was more similar to R-TL than to S-Cairns was on Chromosome 3 in the vicinity of the VGSC gene (315.93–316.4 Mbp) (Fig. 2c). Collectively, VGSC genotyping and genome-wide analyses indicate that the backcrossing procedure with continuous selection for resistance to permethrin, resulted in the introduction of kdr alleles into the susceptible genomic background (R-BC had 100% homozygosity for the resistant parental kdr alleles, and > 99.9% genome of the susceptible origin) and higher level of genome-wide homozygosity.

**Table 3:** *F*_ST_ values averaged across the entire genome-wide, and per each chromosome

Dataset	Total	Chromosome 1	Chromosome 2	Chromosome 3
R-BC vs R-TL	0.237	0.216	0.226	0.269
R-BC vs S-Cairns	0.098	0.082	0.080	0.131
R-TL vs S-Cairns	0.103	0.096	0.104	0.107

### Fitness effects of kdr-driven resistance in *A. aegypti* strains

#### Fecundity

The proportion of female mosquitoes that failed to lay a single egg was not significantly different between the R-BC and the susceptible parental strain, S-Cairns (*χ*^2^ = 0.058, *df* = 1, *P* = 0.9), but it was significantly higher than the resistant parental strain R-TL (*χ*^2^ = 6.48, *df* = 1, *P* = 0.011) (Table 4). In the event that a female mosquito did lay eggs (egg count ≥ 1), the median number of eggs per female in the R-BC was intermediate to the parental strains and not significantly different from either (Mann-Whitney R-BC vs S-Cairns *P* = 0.051; R-BC vs R-TL *P* = 0.093). The reduction in fecundity in the backcrossed R-BC strain when compared to the resistant parental strain, that was similar to the susceptible parental strain, suggests that lower fecundity is not related to a pleiotropic effect of kdr, but rather to an unknown mechanism within the R-TL genotype that has been lost during the backcrossing process.

**Table 4:** Fecundity. Percentage (Mean±SE) of females that did not lay eggs, and the median number of eggs per female (non-zero data only; range of median from each replicate)

	S-Cairns	R-TL	R-BC
% females with zero eggs	24.44 ± 9.7	6.67 ± 3.8	26.67 ± 6.7
Median no. of eggs (non-zero data only)	59 (48–68)	71 (62–87.5)	66 (55–86)

Data obtained from three replicates of 15 females from each of the three strains

#### Larval development time and viability

**Fig. 3:**
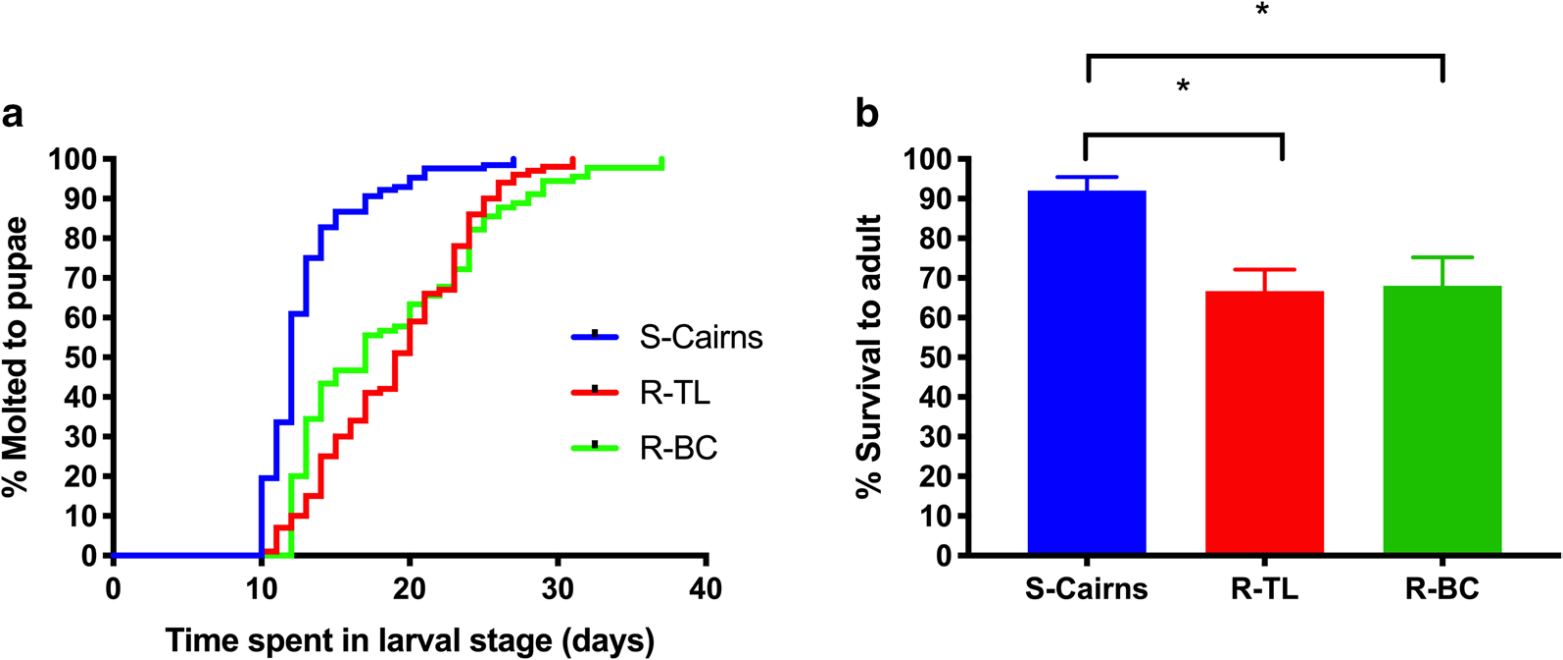
**a** Survival curves (Log-rank (Mantel-Cox) test) for larval development time. **b** Percent larval survival to adult (Mean ± SE). *P < 0.05

The median number of days from first-instar larvae through to pupation in the backcross R-BC strain was 17 days and was not significantly different from the resistant parental strain, R-TL (19 days, Mann-Whitney *U* = 4034.5, *P* = 0.109), but it was significantly higher than in the susceptible parental strain, S-Cairns (12 days, Mann-Whitney *U* = 2263, *P* < 0.001). This was also shown using survival curves where larvae from the R-BC strain took longer to develop to pupae than larvae from the S-Cairns strain (*χ*^2^ = 61.31, *df* = 1, *P* < 0.0001) but was not significantly different to the R-TL strain (*χ*^2^  = 0.16, *df* = 1, *P* = 0.6888) (Fig. 3a). Moreover, a smaller proportion of insecticide resistant larvae emerged as adults when compared to larvae from the S-Cairns strain (R-BC: *χ*^2^ = 7.862, *df* = 1, *P* = 0.005; R-TL: *χ*^2^ = 10.19, *df* = 1, *P* = 0.0014) (Fig. 3b), but there was no significant difference among the three strains in the proportion of females that emerged (*χ*^2^ = 2.824, *P* = 0.244). The differences in larval development between R-BC and S-Cairns suggests a pleiotropic effect of the isolated kdr genotype.

#### Adult longevity

**Fig. 4:**
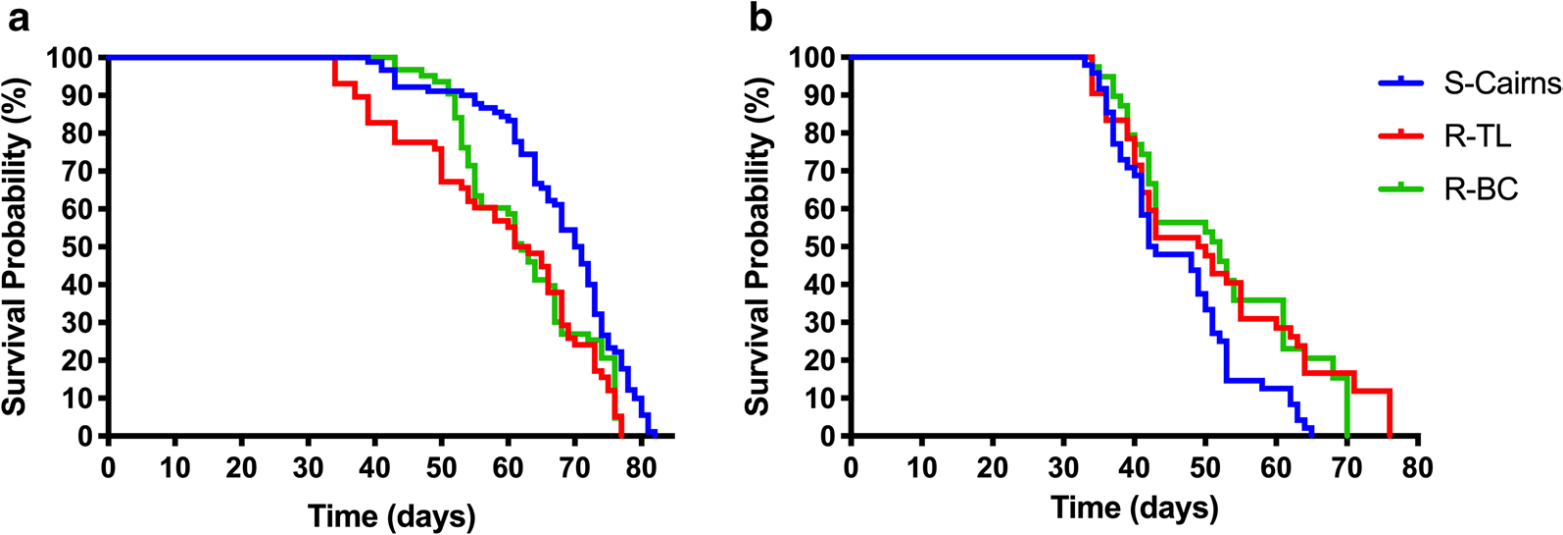
Adult survival curves (Log-rank (Mantel-Cox) test) for caged females (**a**) and males (**b**) of each strain of *Aedes aegypti*

**Fig. 5:**
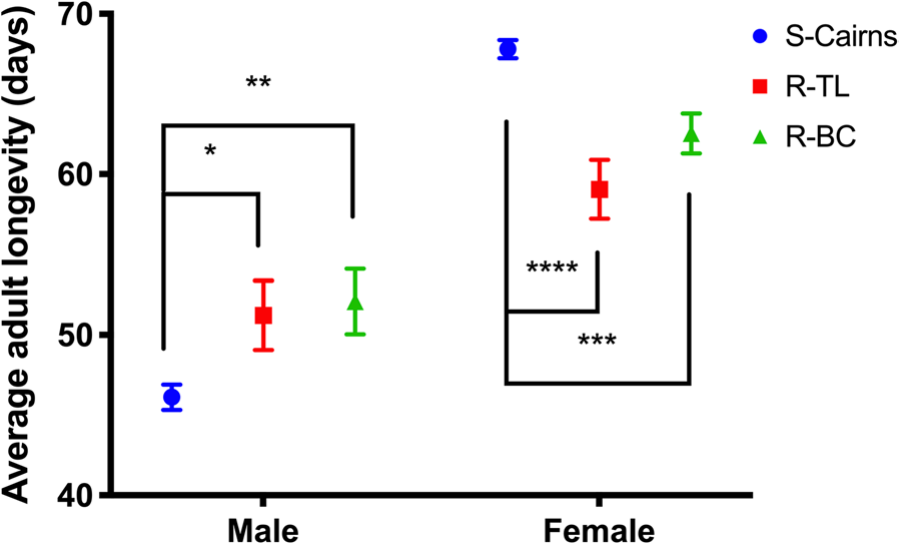
Average adult longevity of males and females from each experimental strain (Mean ± SE). *P < 0.05, **P < 0.001, ***P < 0.0001, ****P < 0.00001

Survival curves generated from the Log-rank (Mantel-Cox) test indicated a decrease in longevity of adult R-BC females, compared to the susceptible S-Cairns strain (*χ*^2^ = 11.92, *df* = 1, *P* = 0.0006). Females from the R-BC and S-Cairns strains showed similar daily survival rates until around day 50 when relative survival declined more quickly in the R-BC strain. Females from the R-TL strain also showed a decrease in longevity in comparison to females from the S-Cairns strain (*χ*^2^ = 15.7, *df* = 1, *P* < 0.0001) however, no significant difference in female longevity was recorded between females from the R-BC and R-TL strains (*χ*^2^ = 1.23, *df* = 1, *P* = 0.2674) (Fig. 4a). The mean survival of females for S-Cairns, R-TL, and R-BC was 68, 59 and 62 days, respectively (Fig. 5). This suggests that reduced longevity is a kdr-related trait in females. In contrast, males from the resistant R-BC and R-TL strains had higher survival rates compared to the susceptible S-Cairns strain (*χ*^2^ = 8.95, *df* = 1, *P* = 0.0028, and *χ* = 6.459, *df* = 1, *P* < 0.011 respectively). No significant difference in mortality was recorded between males from the R-BC and R-TL strains (*χ*^2^ = 0.097, *df* = 1, *P* = 0.7554) (Fig. 4b). In all strains, male survival was reduced in comparison to females (46, 51 and 52 days for S-Cairns, R-TL and R-BC strains, respectively) (Fig 5).

#### Mosquito size

**Fig. 6:**
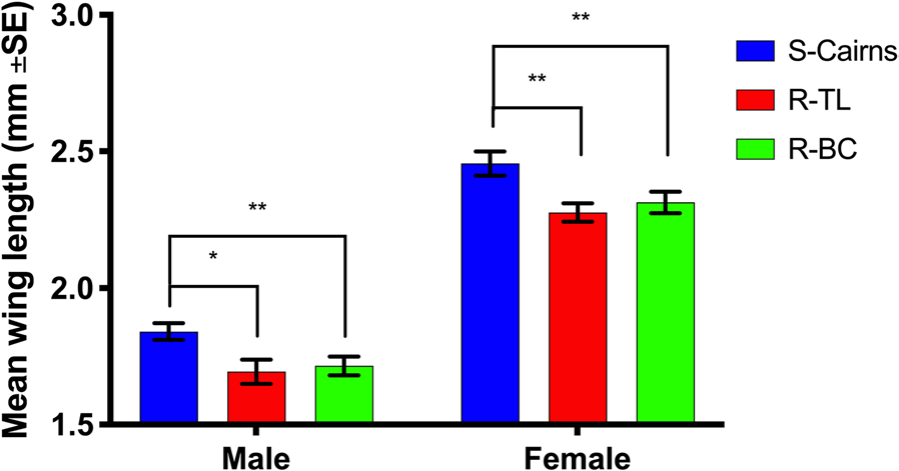
Average wing length (mm) as a proxy for body size in S-Cairns, R-TL and R-BC *Aedes aegypti* males and females (Mean±SE). *P<0.05, **P<0.001

Calculation of wing length were used as a proxy for body size, and this indicated a smaller size for mosquitoes from both resistant strains when compared to the susceptible strain. This was true for females (R-BC vs S-Cairns females, *t*_(96)_ = 2.391, *P* = 0.0187; R-TL vs S-Cairns females, *t*_(100)_ = 3.239, *P* = 0.0016) and males (R-BC vs S-Cairns males, *t*_(97)_ = 2.757, *P* = 0.007; R-TL vs S-Cairns males, *t*_(96)_ = 2.759, *P* = 0.0069, Fig. 6). From these results, it appears that smaller body size is a pleiotropic effect of kdr.

## Discussion

The evolution and selection of insecticide resistance in insect pests have long been assumed to have associated fitness costs [34]. That hypothesis is central to resistant management strategies in agriculture and pertinent to the wider discussion around how to preserve insecticide susceptibility in mosquito vectors of disease [35, 36]. In *Aedes aegypti*, the global vector of dengue, chikungunya, Zika and yellow fever, insecticide resistance is becoming ubiquitous, particularly concerning several point mutations at the pyrethroid target site, the sodium channel [37]. These are collectively termed kdr mutations. It is commonly held that investment in these resistance mechanisms affect other important biological processes and are associated with a loss of fitness in the absence of insecticides [3, 8, 19, 38, 39]. However, with few exceptions [3], the studies that measure such costs are rarely definitive because they are seldom performed on genetically related strains with isolated resistant alleles. In this report, we assessed the pleiotropic effects of a double homozygous resistant kdr genotype (V1016G/S989P) in *A. aegypti* by backcrossing those kdr alleles into an otherwise susceptible genetic background.

To the best of our knowledge, this is the first study on insecticide resistance in *A. aegypti* to use a reduced genome sequencing to confirm the genetic constitution of our backcrossed line and isolation of a target insecticide-resistant allele. Thus, differences in life-history traits between the resistant backcrossed strain and the susceptible strain used in this study can be attributed mainly to a pleiotropic effect of the isolated kdr mutations and nearby variants in high linkage disequilibrium. Bioassays confirmed that our backcross, R-BC strain, had a similar resistance profile to the resistant parent, the R-TL strain. The only significant difference between their phenotypes was that, at the highest doses, R-BC was not as resistant as R-TL. This may indicate the loss of additional protective alleles not related to kdr, as well as some fitness cost from increased genome-wide homozygosity in R-BC individuals.

Our study revealed that kdr mutations, V1016G and S989P confer a high level of resistance to type I pyrethroids (permethrin and lambda-cyhalothrin) and low-level resistance to deltamethrin (type II pyrethroid) in our *A. aegypti* strain. The function of V1016G has previously been demonstrated using an *in vitro* expression system [11] and is known to affect the action of both permethrin and deltamethrin [10]. The S989P mutation was linked with the V1016G mutation in our pyrethroid-resistant strains, but it has not been found to have any neurophysiological effect in isolation [11]. Ours is the first study to have identified the costs of the double V1016G/S989P genotype in any insect, although, in a previous study, Brito et al. did detail the costs of an isolated V1016I/F1534C genotype in a backcrossed *A. aegypti* strain [3]. Interestingly, V1016I alone has no impact on pyrethroid sensitivity and its role in promoting or ameliorating fitness costs remains unclear [10, 40]. Therefore, the reported increase in larval development time, a reduction in fecundity and poorer competition in caged populations during the study may be linked to a pleiotropic effect of the F1534C mutation, which does have a functional role in insecticide resistance.

In our study, the R-BC strain exhibited longer larval development times (5 days), 24% fewer mosquitoes reached the adult stage, had smaller wing lengths (females and males) and female mosquitoes had a shorter lifespan (6 days), when compared to the susceptible strain. We did not detect differences in the number of eggs laid, indicating that these fitness parameters cannot be attributed to the pleiotropic effect of the kdr mutations. In a previous study, the V1016G/S989P genotype was not associated with any change in fertility for *A. aegypti* from Thailand when compared to a strain without kdr mutations from the same province. The same study did document a decrease in fertility associated with the F1534C mutation [4], but it is important to note that in these cases, kdr mutations were not isolated from otherwise comparable genetic backgrounds, thereby preventing distinction between true and incidental associations between kdr and reproductive costs in *A. aegypti*.

In the field, the larval stage is arguably the most important time in the mosquito life-cycle as it is the stage that accumulates nutritional reserves [41]. We evaluated average larval development time and the success rate at which first instar larvae progressed to the adult stage. Both of these parameters were negatively affected by the isolated kdr mutations in the R-BC strain. A significant increase in larval development time has been previously observed in insecticide-resistant *A. aegypti* with the V1016G and S989P kdr mutations [4], but as discussed above, that increase cannot be directly attributed to the kdr mutations.

Delays in larval development in individuals with the double homozygous kdr genotype (V1016G/S989P) may be due to a requirement for additional nutritional reserves to offset the energetic costs of harbouring kdr mutations. This may increase the risk of parasitism and predators in the larval environment [42]. Male *A. aegypti* develop faster than females [43]. A delay in male larval development time, and slower emergence from the aquatic habitat, might reduce their opportunities to mate with females when compared to their faster-developing competitors. This is especially important when considering that female mosquitoes do not usually mate more than once [44]. Delayed larval development time in males may, therefore, confer a competitive disadvantage to the V1016G/S989P mutation in an insecticide-free environment.

The extrinsic incubation period (EIP) is the time required for a mosquito to become infectious to a mammalian host after that mosquito has imbibed a blood meal containing a mosquito-borne pathogen. The EIP in *A. aegypti* is reported as 8–12 days [45, 46] for the dengue virus, 10 days for the Zika virus [47] and 6–8 days for chikungunya virus [48]. Reductions in adult mosquito longevity can, therefore, have significant effects on the transmission of vector-borne disease [46, 49]. Additionally, a decrease in longevity could decrease vector abundance due to a reduction in gonotrophic cycles and subsequently, a reduction in egg production [50]. We observed a 6-day reduction in adult female R-BC longevity, from an average adult lifespan of 68 days in the S-Cairns strain. This extremely extended laboratory-observed survival clearly bears little relation to field estimates of longevity but, if it implies a general reduction in the age of field populations, then kdr may affect disease transmission. In contrast to females, males from our insecticide-resistant strains had higher adult longevity in comparison to insecticide susceptible males (5 and 6 days increase in longevity for R-TL and R-BC, respectively). The increase in male longevity that we observed could be a result of the extended larval stage in males with kdr mutations, allowing for a greater accumulation of nutritional resources [51]. It could be expected that such a process also leads to greater body size of resistant males, but this was not observed in our resistant strains. Nevertheless, our results suggest that kdr-driven changes in some life-history traits are sex-specific.

We also found that both males and females from the resistant strains (R-BC and R-TL) had smaller wing length when compared to the insecticide susceptible S-Cairns strain. Wing length has been used as a proxy for body size [52, 53], a trait that is one of the most significant predictors of physiological fitness in the observed interactions between the environment and life-history traits. Larger mosquitoes have been reported as being more tolerant to insecticides [54], having enhanced host-seeking behaviours [55] and greater male reproductive capacity as measured by spermatozoa number [56], although larger males might not always be the most successful in finding partners during mating [57]. Our observation that smaller body size is linked to a kdr genotype might suggest another potential disadvantage to the V1016G/S989P genotype. Reductions in *A. aegypti* female wing length have previously been associated with the same mutations but without the certainty afforded by a comparison of strains with otherwise identical genetic backgrounds [4].

Our backcrossing protocol involved the application of insecticide selection pressure at every generation. This resulted in a homozygous resistant strain, similar to the resistant parent, a genotype that is common across Asia [1, 58, 59]. We did not examine the costs associated with heterozygous resistant kdr forms. Heterozygotes typically display responses to insecticides that are intermediate between homozygous susceptible and resistant strains [46], but limited studies of their pleiotropic impacts suggest that they often align with susceptible genotypes [60, 61].

The identification of the pleiotropic impacts of specific kdr mutations under carefully controlled laboratory conditions has value, in that meticulous backcrossing programs remain the principal way to explore these associations [62], but it is impossible to know how these costs might manifest themselves in field settings and in epistatic interaction with a multitude of genotypes. Nonetheless, such careful laboratory evaluations are an essential step in understanding that some kdr mutations do have deleterious pleiotropic effects. This helps to support the adoption of resistant management strategies that aim to reduce selection pressure and return competitive advantage to susceptible insects. For *A. aegypti*, there are no well-documented examples of successful resistant management strategies, although the reactive substitution of pyrethroids with other insecticide classes is common [37]. Still, it is interesting to note that Grossman *et al.* recently showed that kdr frequencies decreased and pyrethroid susceptibility was restored to highly resistant strains of *A. aegypti* maintained in semi-natural conditions in the absence of insecticide [63]. These kinds of proofs, in tandem with careful laboratory studies such as ours, support a proactive vector control strategy that uses the full range of available insecticide classes in rotations, mosaics, or temporal refugia to reduce selection pressures and encourage reversion to susceptibility.

## Conclusions

Vector control programs that utilise mosaics or rotations of insecticides with different modes of action, can be particularly effective if there are fitness costs associated with resistance mechanisms [64]. Our study, comparing parental and backcrossed strains, provides evidence that the V1016G/S989P genotype, in an otherwise susceptible genetic background, has pleiotropic effects on body size, larval development, and adult longevity. This gives empirical support for the implementation of vector control and resistance management strategies that reduce pyrethroid selection pressure in the field and permit susceptible mosquitoes to reassert their competitive advantage.

## Supplementary information

**Additional file 1: Figure S1.** Backcrossing method. Lab-bred female *Aedes aegypti* originally from Timor-Leste are crossed with males from Cairns, Australia. F1 hybrids are backcrossed to S-Cairns males post-selection for insecticide resistance (IR) with a DD of permethrin. This procedure continued to F12 after which time the surviving progeny were allowed to mate freely and were maintained as a single colony at each generation.

**Additional file 2: Figure S2.** Bioassay results for permethrin selection during the creation of strain R-BC (Mean ± SE).

**Additional file 3: Table S1.** Total SNPs for each dataset used in heterozygosity and *F*_ST_ analysis.

## Data Availability

Data supporting the conclusions of this article are included within the article and its Additional files 1, 2, 3. The datasets used and analysed during the current study are available from the corresponding author on reasonable request.
