# Supplementary material for: Identifying the fitness costs of a pyrethroid-resistant genotype in the major arboviral vector Aedes aegypti
Source: Parasit Vectors. 2020 Jul 20;13:358. doi: 10.1186/s13071-020-04238-4 (PMC7372837; doi:10.1186/s13071-020-04238-4)
Supplement: Supplementary file 1 — Additional file 1: Figure S1. Backcrossing method. Lab-bred female Aedes aegypti originally from Timor-Leste are crossed with males from Cairns, Australia. F1 hybrids are backcrossed to S-Cairns males post-selection for insecticide resistance (IR) with a DD of permethrin. This procedure continued to F12 after which time the surviving progeny were allowed to mate freely and were maintained as a single colony at each generation. [file 13071_2020_4238_MOESM1_ESM.pdf]

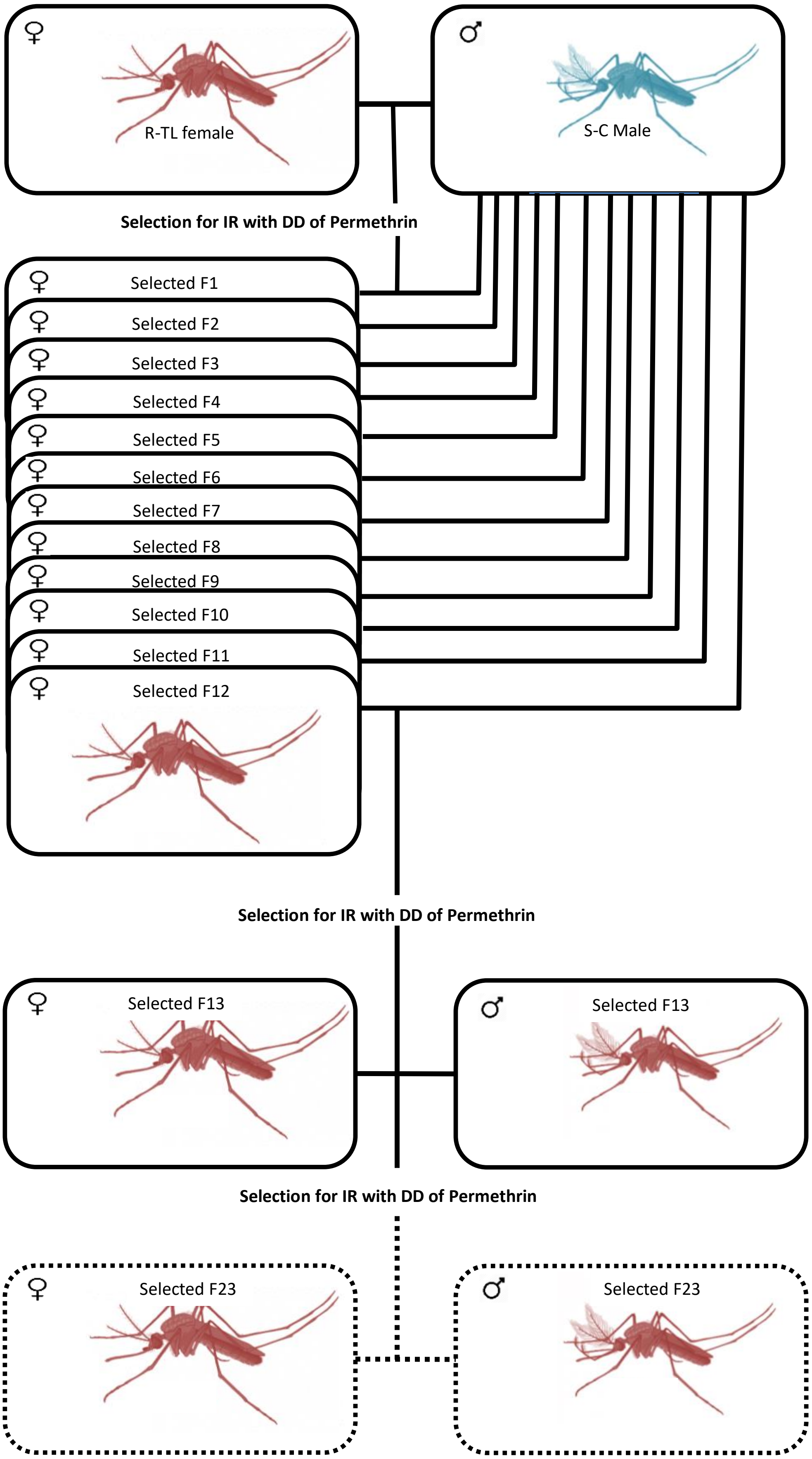

♀
R-TL female
♂
S-C Male
Selection for IR with DD of Permethrin
Selected F1
Selected F2
Selected F3
Selected F4
Selected F5
Selected F6
Selected F7
Selected F8
Selected F9
Selected F10
Selected F11
Selected F12
Selection for IR with DD of Permethrin
Selected F13
Selected F13
Selection for IR with DD of Permethrin
Selected F23
Selected F23
