# Supplementary figures and images for: Identifying the fitness costs of a pyrethroid-resistant genotype in the major arboviral vector Aedes aegypti
Source: Parasit Vectors. 2020 Jul 20;13:358. doi: 10.1186/s13071-020-04238-4 (PMC7372837; doi:10.1186/s13071-020-04238-4)

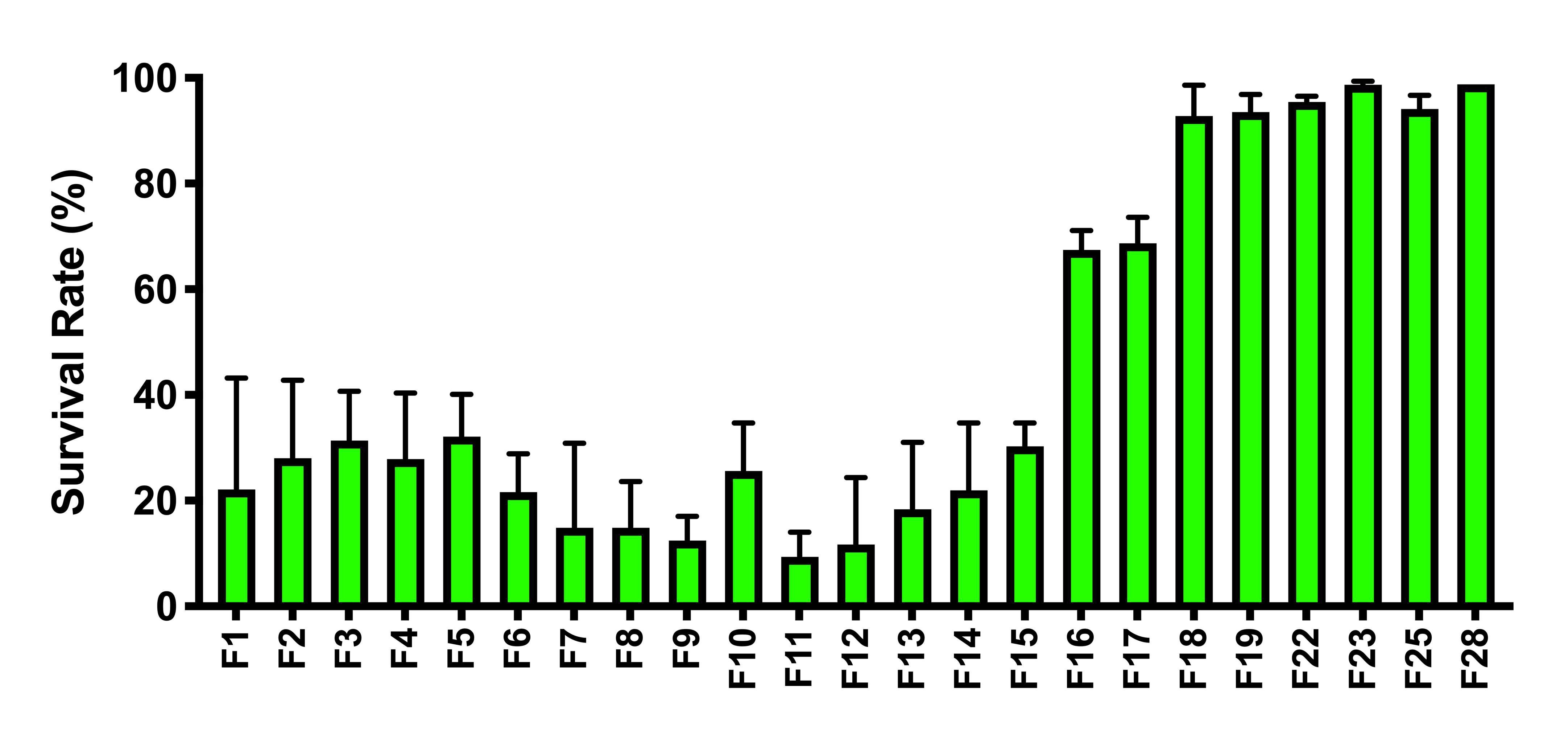

Supplement: Supplementary file 2 — Additional file 2: Figure S2. Bioassay results for permethrin selection during the creation of strain R-BC (Mean ± SE). [file 13071_2020_4238_MOESM2_ESM.tiff]
