# Supplementary material for: Identifying the fitness costs of a pyrethroid-resistant genotype in the major arboviral vector Aedes aegypti
Source: Parasit Vectors. 2020 Jul 20;13:358. doi: 10.1186/s13071-020-04238-4 (PMC7372837; doi:10.1186/s13071-020-04238-4)
Supplement: Supplementary file 3 — Additional file 3: Table S1. Total SNPs for each dataset used in heterozygosity and FST analysis. [file 13071_2020_4238_MOESM3_ESM.docx]

| **Dataset** | **Total number of SNPs** | **Chromosome 1** | **Chromosome 2** | **Chromosome 3** |
| --- | --- | --- | --- | --- |
| R-TL and R-BC | 18510 | 4673 | 7894 | 5943 |
| R-BC and S-Cairns | 23903 | 5816 | 9898 | 8189 |
| R-TL and S-Cairns | 25833 | 6276 | 10693 | 8863 |
